# Extreme Anharmonicity and Thermal Contraction of One-Dimensional Wires

**DOI:** 10.1021/acs.nanolett.5c04282

**Published:** 2025-10-27

**Authors:** Chiara Cignarella, Lorenzo Bastonero, Lorenzo Monacelli, Nicola Marzari

**Affiliations:** † Theory and Simulation of Materials (THEOS) and National Centre for Computational Design and Discovery of Novel Materials (MARVEL), 27218 École Polytechnique Fédérale de Lausanne, 1015 Lausanne, Switzerland; ‡ U Bremen Excellence Chair, Bremen Center for Computational Materials Science, and MAPEX Center for Materials and Processes, 9168 University of Bremen, 28359 Bremen, Germany; ¶ Department of Physics, 9311 Sapienza University of Rome, 00185 Rome, Italy; § PSI Center for Scientific Computing, Theory, and Data, Laboratory for Materials Simulations, Paul Scherrer Institut, 5232 Villigen, Switzerland

**Keywords:** one-dimensional materials, stochastic self-consistent harmonic approximation, anharmonicity, heat capacity, negative thermal expansion

## Abstract

Ultrathin nanowires could play a central role in next-generation downscaled electronics. Here, we explore some of the most promising candidates identified from previous high-throughput screening—CuC_2_, TaSe_3_, and AuSe_2_—to gain insight into the thermodynamic and anharmonic behaviors of nanowires that could be exfoliated from weakly bonded three-dimensional materials. We analyze thermal stability, linear thermal expansion, and anharmonic heat capacity using the stochastic self-consistent harmonic approximation. Notably, our work unveils exotic features common among all one-dimensional wires: a colossal record negative thermal expansion and very large deviations from the Dulong–Petit law due to strong anharmonicity.

## Graphical abstract

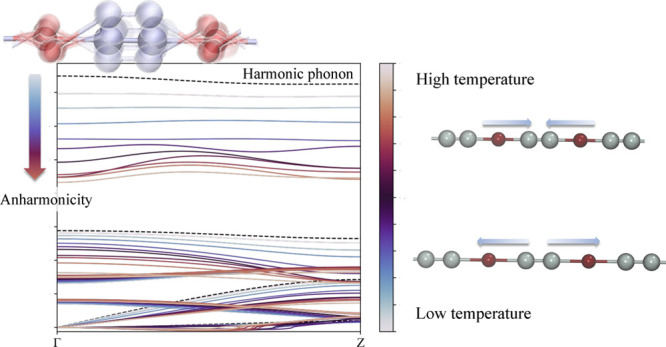

The great progress of nanotechnology has pushed the exploration of one-dimensional (1D) materials.^1,2^ Since the first synthesis of carbon nanotubes (CNTs),^3–6^ which, despite their unique properties, have faced difficulty in controlled synthesis, research has made significant progress, achieving the realization of ultrathin nanowires with few-atom diameters or even single-atom chains.^7,8^ In fact, 1D materials could be highly beneficial in the continuous downscaling of next-generation electronic devices^9,10^ because they naturally lack grain boundaries and edge scattering, standing out as ideal candidates for nanoelectronic applications.^11–13^ Equally important, they represent a new playground of fundamental physical phenomena, such as Luttinger liquid, Peierls transition, and excitonic insulators.^14–17^ Various techniques have been explored to produce 1D crystals, including self-assembly and direct growth on substrates,^18–20^ directing-agents synthesis,^7^ encapsulation inside single-walled or multiwalled CNTs,^21,22^ or chemical/mechanical exfoliation from three-dimensional (3D) crystals.^11,23^ The latter represents a promising direction to obtain novel wires with the desired and well-defined properties. Alongside, computational high-throughput (HT) studies can search for bulk crystals potentially exfoliable into single nanowires,^24–27^ i.e., materials naturally composed by strongly bonded inorganic wires held together by van der Waals interactions, in a fashion similar to what is done for two-dimensional (2D) materials.^28–31^ In this context, a HT search^26^ has been conducted to discover novel 1D nanowires that could be exfoliated from experimentally known and previously synthesized crystals. From the resulting database of more than 800 unique 1D wires, a recent work focused on metallic wires^27^ searching for materials resilient to dynamical instabilities, such as Peierls distortions, that could represent alternatives as interconnects for future downscaled electronic devices.

In this work, we aimed to gain a comprehensive understanding of the thermodynamics of exfoliable wires for nanotechnologies. We selected three wires, CuC_2_, TaSe_3_, and AuSe_2_, from the previous database. CuC_2_ and TaSe_3_ are two metallic wires stable at *T* = 0 K.^27^ CuC_2_ is the thinnest metallic wire predicted to be exfoliable from 3D bulks;^27^ it is a straight-line chain stable at 0 K when exfoliated from the bulk parent structure. The wire has the highest Young’s modulus within the previous portfolio, it is bendable while maintaining the metallic state, and it is robust against O_2_ oxidation or contamination.^27^ Among these materials, TaSe_3_ was already exfoliated experimentally in 2016 ^11^ and, shown to be a remarkably good candidate for downscaled applications, was proposed as an alternative for local interconnects in field-effect transistors (FET).^12,32^ AuSe_2_, on the other hand, is unstable in its exfoliated metallic phase at 0 K, but it presents a stable semiconducting phase in a double-cell superstructure.^27^ It is a candidate for the realization of Peierls transition and relevant for applications in switches or multifunctional devices, where the metal–insulator transition can be tuned by temperatures, mechanical strains, or electric fields.^33–35^

All of these materials are susceptible to charge-density-wave (CDW)-like fluctuations even in the stable phase, which are typically accompanied by strong anharmonicity at finite temperature, relevant for practical applications. The standard computational approach to deal with anharmonicity is molecular dynamics, which, however, does not include quantum effects of the nuclei. Those can play an important role, for example, in CuC_2_ due to the presence of strong covalent bonds and light carbon atoms, leading to a Debye temperature above 1000 K. Capturing the quantum thermodynamics of crystals beyond the harmonic approximation from first principles requires complex simulations, such as those based on path-integral molecular dynamics.^36^ However, these techniques may become computationally very expensive, particularly when multiple materials need to be analyzed. Recently, the stochastic self-consistent harmonic approximation (SSCHA)^37,38^ has emerged as a state-of-the-art method to simulate materials beyond the harmonic approximation,^39–43^ accounting for both quantum and thermal ionic fluctuations, while computing properties at different temperatures. Here, for the first time, we extensively study the thermodynamics of realistic 1D materials with a full treatment of anharmonicity. The SSCHA^37,38^ minimizes the vibrational quantum free energy by optimizing a trial Gaussian ionic density matrix ρ̃(**R**).

Nevertheless, the application of this approach to 1D systems presents several challenges arising from their inherent low-frequency phonon bands. Real 1D systems exhibit four acoustic phonon modes instead of the three given by translational invariance: 1D crystals are, in fact, also invariant to rotational modes around the wire axis;^44^ notably, this feature is not present in perfect in-line chain-like carbyne,^40^ where the rotation around the 1D axis is an identity. On these 1D materials, accounting for the rotational mode is essential to remove the extra fourth acoustic zero frequency at Γ that makes the free energy minimization extremely unstable (and leads to nonphysical results). For the purpose of this work, we lock the rotation around the wire axis, effectively implementing the acoustic sum rule (ASR) for 1D materials in the SSCHA framework.^45^

In addition, among the four acoustic phonons, 1D crystals exhibit two *flexural* modes that are quadratic in the long-range limit [ω­(*q*) ∼ *q* ^2^] and, therefore, have very low frequencies in a broad region of the Brillouin zone. The low-frequency nature of these modes requires a high number of configurations to sample the small forces acting on them (or, alternatively, the large atomic displacements associated with them),^46^ which makes full ab initio SSCHA calculations prohibitive. To address this limitation, we train machine-learning interatomic potentials for our systems, which allow us to compute thousands of configurations within a reasonable time and computational cost, in particular, using an equivariant neural network architecture, as implemented in the open-source NequIP code^47^ (more details can be found in the Supporting Information, SI).

Understanding how the structure of nanowires reacts to temperature is fundamental for real applications of 1D wires in downscaled electronics. Low-dimensional materials show contraction when heated.^48,49^ To study the behavior of nanowires upon heating, we investigated thermal expansion. That can be described by the linear expansion coefficient α, which characterizes how the length of the wire changes with temperature. We exploited well-known thermodynamic relationships to determine the thermal expansion from simulations performed at fixed volume:^42^

1 $$\frac{\alpha }{\beta_{T}}={\left(\frac{\partial P}{\partial T}\right)}_{V}$$

where β_T_ is the isothermal compressibility, i.e., the inverse of the bulk modulus, and the subscript *V* indicates that the derivative is performed at constant volume. In 1D materials, the volumetric thermal expansion coefficient is equivalent to the linear thermal expansion coefficient because expansion only occurs along the axial direction of the wire, and we use eq 1, with the Young’s modulus *Y* taking the role of the bulk modulus (more details are given in the SI).

To assess the degree of anharmonicity, we compute α with both the SSCHA and the quasi-harmonic approximation (QHA).^50,51^ The QHA has been successfully applied in many systems to study the thermal expansion and temperature-dependent vibrational properties.^46,48,52^ However, QHA maintains the harmonic assumption of temperature-independent and noninteracting phonon frequencies. The role of anharmonicity is accounted for by assuming that phonon frequencies depend only on volume or, in the case of 1D crystals, on the tensile strain. This approximation falls short when anharmonicity is significant, such as at high temperatures.^52^ Nevertheless, it provides qualitative insight into thermal expansion because α­(*T*) can be decoupled into the individual contribution from each phonon mode.

At constant volume, the heat capacity *C*_*V*_ is defined as the derivative of the internal energy *U* with temperature ${\left(\frac{\partial U}{\partial T}\right)}_{V}\equiv T{\left(\frac{\partial S}{\partial T}\right)}_{V}$ and quantifies the amount of energy required to raise the temperature of the system. When anharmonicity is taken into account, phonon frequencies change with temperature even at constant volume, introducing an additional dependence of entropy *S* to temperature through phonon frequencies, which corrects the result of the harmonic theory:^42^

2 $$C_{V}=T\;\frac{\partial S}{\partial T}+T\underset{q,\mu }{\sum }\frac{\partial S}{\partial \omega_{q,\mu }}\frac{\partial \omega_{q,\mu }}{\partial T}=\underset{q,\mu }{\sum }C_{V}^{\mathrm{harm}}\left(q,\mu \right)\left(1+\frac{T}{\omega_{q,\mu }}\frac{\partial \omega_{q,\mu }}{\partial T}\right)$$

The first term is the heat capacity of a perfect harmonic system:

3 $$C_{V}^{\mathrm{harm}}\left(q,\mu \right)=k_{B}{\left(\frac{\hbar \omega_{q,\mu }}{k_{B}T}\right)}^{2}\frac{e^{\hbar \omega_{q,\mu }/k_{B}T}}{{\left(e^{\hbar \omega_{q,\mu }/k_{B}T}-1\right)}^{2}}$$

while the second accounts for the intrinsic anharmonic changes of frequencies with temperature when the volume is fixed. In the high-temperature limit, the harmonic contribution to the total heat capacity recovers the classical Dulong–Petit law, which becomes temperature-independent when all vibrational degrees of freedom are thermally active:

4 $$\underset{T\to \infty }{\lim}\underset{q,\mu }{\sum }C_{V}^{\mathrm{harm}}\left(q,\mu \right)=3Nk_{B}$$

This is no longer true in strongly anharmonic crystals because phonon frequencies may change with temperature at constant volume, even when all vibrations are thermally excited.

We start with the discussion investigating the thermodynamic properties of CuC_2_. CuC_2_ displays strongly temperature-dependent phonon dispersions, reported in Figure 1a, which are a clear signature of anharmonicity on the structural and thermodynamic properties. To analyze the stability with temperature, we define the positional free energy, which is the SSCHA free energy constraining the average ionic positions (see the SI). The calculation of the temperature-dependent anharmonic phonons from the Hessian of the positional free energy^53^ shows that the wire is stable in its exfoliated configuration across the entire temperature range considered, up to 2000 K, without showing any signal of structural phase transition or emerging instabilities. (All phonon frequencies are positive; note that the small wings around Γ are due to interpolation artifacts.^44^) Notably, the SSCHA frequencies at *T* = 0 K differ from those of the harmonic phonons, indicating a significant renormalization of phonon frequencies due to the quantum zero-point motion (ZPM) in CuC_2_.

**Figure 1:**
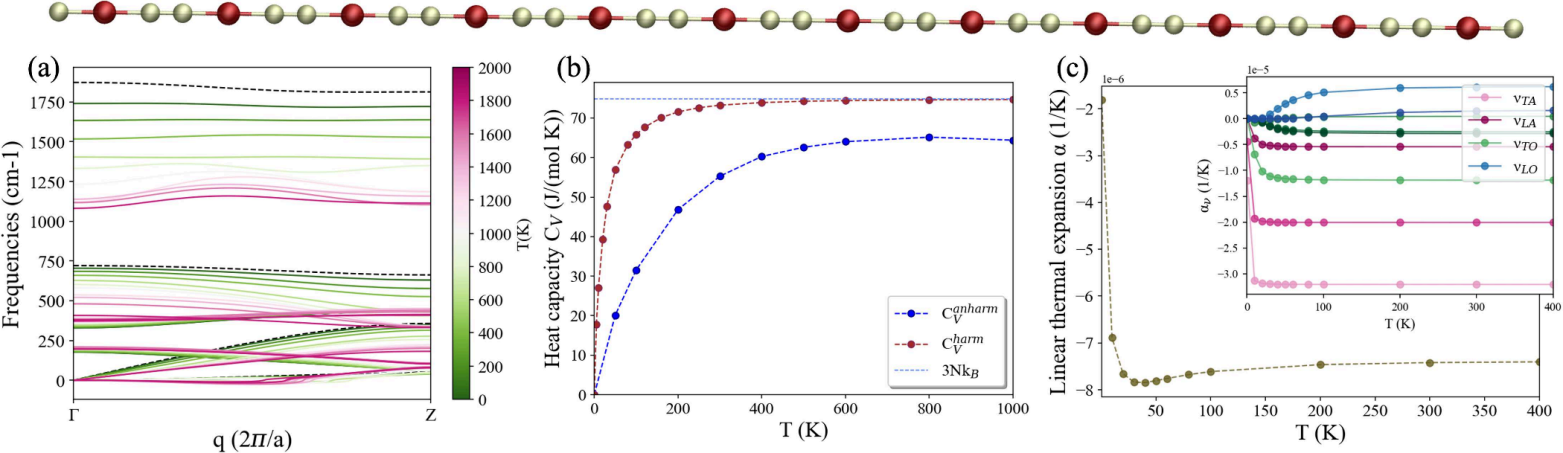
CuC_2_ (Cu, red; C, gray). (a) Phonon dispersions (Hessian of the positional free energy^53^) at different temperatures within the SSCHA for the wire in the configuration, as exfoliated from the 3D wire. Dashed black lines represent harmonic phonons from density functional perturbation theory (DFPT). (b) Anharmonic heat capacity calculated within the SSCHA with eq 2 (blue line) and harmonic heat capacity (brown line) using eq 3 with temperature-independent harmonic DFPT ω. (c) Linear thermal expansion coefficient α­(*T*) using the QHA. In the inset, α­(*T*) per phonon branch. A and O refer to acoustic and optical modes and T and L to transversal and longitudinal modes. Within the same group of modes, lighter colors correspond to lower frequencies.

In Figure 1b, we report the comparison between the specific heat at the SSCHA and the harmonic level: *C*_*V*_ was computed with the correct treatment of anharmonicity using eq 2 (blue line), and *C*_*V*_^harm^ was computed using eq 3 (brown line) with the harmonic frequencies at *T* = 0 K. We observe that the two quantities differ even at low temperature, indicating a strong anharmonic contribution in this system. *C*_*V*_^harm^ reaches the Dulong–Petit limit (the light blue dashed line in the figure) at nearly 200 K, while *C*_V_^anharm^ lies far below this limit at the same temperature and eventually never reaches it. To further analyze the role of the anharmonicity, in the SI, we report and discuss the high-temperature limit of the heat capacity. We note a nonlinear variation of the frequencies upon heating, entirely due to anharmonic effects, which does not saturate at the Dulong–Petit value, showing instead a tendency to decrease at high *T*. This unusual feature highlights the system’s intrinsically strong anharmonicity.

We computed the linear thermal expansion coefficient with the SSCHA (see Figure 4 and the SI): α_CuC_2__ has negative values, with a minimum of (−7.07 ± 0.39) × 10^–5^ K^–1^. CuC_2_ shows a contraction in its length as the temperature increases. Thermal expansion or contraction results from the interplay between the vibrational modes in the crystal. Low-dimensional materials exhibit more freedom for out-of-plane vibrations; they can expand in the vacuum directions with a relatively low energy cost due to their reduced dimensionality. These low-frequency traverse modes dominate at low temperature and can lead to an overall contraction in the longitudinal plane.^54–56^ However, as the temperature rises, excitation of the high-frequency vibrations along the longitudinal axis competes with softer ones, causing the material to expand. Negative thermal expansion (NTE) has been observed in single-walled CNTs, quasi-1D polymers, nanowires,^49,57–59^ or 2D monolayers such as graphene.^48,52,60^ Both graphene and CNTs exhibit a linear thermal expansion coefficient on the order of ∼−1 × 10^–6^ K^–1^, with CNTs reaching its maximum contraction value of −1.2 × 10 ^–5^ K^–1^ at room temperature.^49^ The crossover occurs around 2000 or 900 K for graphene or CNTs, respectively, where α­(*T*) becomes positive and the systems begin to expand.

Figure 1c reports the computed values of α­(*T*) for CuC_2_ using the QHA. Like the fully anharmonic SSCHA result, the QHA also yields negative in-line thermal expansion. However, the quantitative result is different due to significant role of anharmonicity, as was already revealed by analysis of the heat capacity.

While the SSCHA is more accurate, the QHA offers interesting insight into the role played by individual phonon bands. The phonon branches contributing to the negative α are mainly related to vibrations on the *x̂*–*ŷ* transversal plane (typically of the two carbon atoms), labeled TA/TO in the inset in Figure 1c, with the first two transversal acoustic modes ν_TA_ and the first optical mode ν_TO_ giving the strongest contributions. Longitudinal modes ν_L_ aligned with the wire axis *ẑ* contribute positively (ν_LO_) or slighlty negatively (ν_LA_) compared to TA modes.

Figure 2a displays the temperature-dependent phonon band structure obtained from the Hessian of the positional free energy^53^ of the single wire of TaSe_3_. This analysis unveils the emergence of instabilities with temperature, manifesting as imaginary phonon frequencies and indicated by the blue arrow in the plot. The system, which is stable at low temperature, acquires an imaginary (negative) acoustic phonon above 650 K, marking the upper critical *T* above which the 1D chain becomes unstable. The instability is located at ∼^3^/_4_ between Γ and *Z* (1,1,1/2)­[2π/*a*] and is due to the movement of selenium atoms along the perpendicular plane to the wire axis (see the SI). This phase transition may represent a novel high-temperature CDW state of TaSe_3_ or thermal melting of the structure, which is likely to occur in real systems at such high temperatures. Notably, the TaSe_3_ wire remains (meta)­stable until 650 K in the exfoliated phase from the 3D-parent compound, with remarkable perspectives for technological applications.

**Figure 2:**
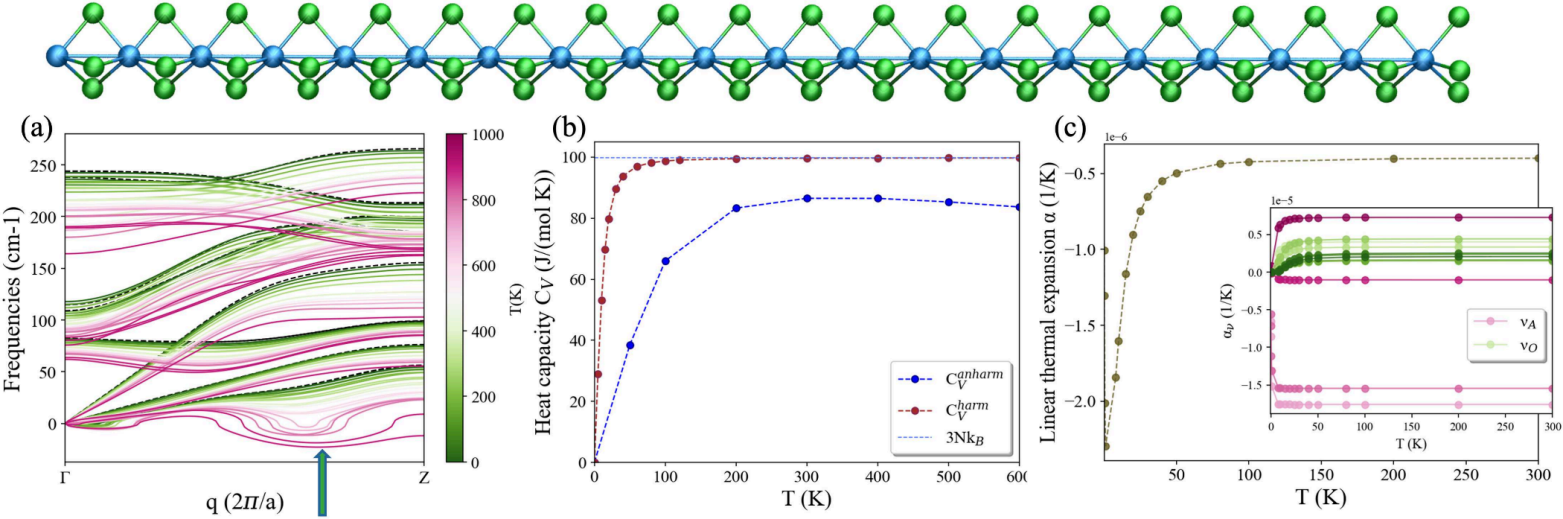
TaSe_3_ (Ta, blue; Se, green). (a) Phonon dispersions (Hessian of the positional free energy) at different temperatures within the SSCHA for TaSe_3_ in the configuration, as exfoliated from 3D wires.^1^ Dashed black lines represent harmonic phonons from DFPT. The blue arrow highlights the unstable modes appearing from 700 K. (b) Anharmonic heat capacity calculated within the SSCHA with eq 2 (blue line) and harmonic heat capacity (brown line) using eq 3 with temperature-independent harmonic DFPT ω. (c) Linear thermal expansion coefficient α­(*T*) using the QHA. In the inset, α­(*T*) per phonon branch. A and O refer to acoustic and optical modes. Within the same group of modes, lighter colors correspond to lower frequencies. Note that the small wings around Γ are interpolation defects and sensitive to the ASR imposition, in contrast to the genuine instability at 700 K, which aligns with an explicit *q* point in the sampling mesh.

In Figure 2b, the comparison between harmonic and anharmonic heat capacities is shown. As before, the anharmonicity of TaSe_3_ manifests in the strong difference between *C*_V_ computed with the SSCHA and harmonic calculations. The high-temperature limit of the heat capacity presented in the SI shows a decreasing trend with the temperature, further underlining the strong anharmonicity.

The SSCHA thermal expansion coefficient computed for TaSe_3_ results in a minimum α = (−1.26 ± 0.07) × 10^–5^ K^–1^ (see Figure 4 and the SI). Also TaSe_3_ exhibits contraction, smaller than CuC_2_ yet comparable with the maximum contraction reported for CNTs,^49^ and other well-known NTE materials. The QHA (Figure 2c) shows that this is mostly given by the first two lowest-frequency acoustic modes (lighter violet), representing symmetric vibrations of the selenium atoms on the *x̂*–*ŷ* plane that produce a bending of the wire; this is followed by the third lowest-frequency acoustic mode, associated with twisting, whereas the longitudinal acoustic mode (darkest violet) yields instead a positive contribution.

We investigate the properties of AuSe_2_ in the reconstructed stable phase with double-cell periodicity,^27^ which is insulating and with a Kohn–Sham band gap at the PBE level *E*_g_ = 0.48 eV.^27^ We inspect the behavior of the band gap with temperature,^61,62^ as illustrated in Figure 3a: notably, *E*_g_ decreases significantly, from 0.48 eV at 0 K (0.43 ± 0.01 eV due to ZPM) to 0.13 ± 0.06 eV at 1000 K. This temperature-dependent band gap can be exploited, for instance, in optoelectronic devices or temperature sensors.^63,64^

**Figure 3:**
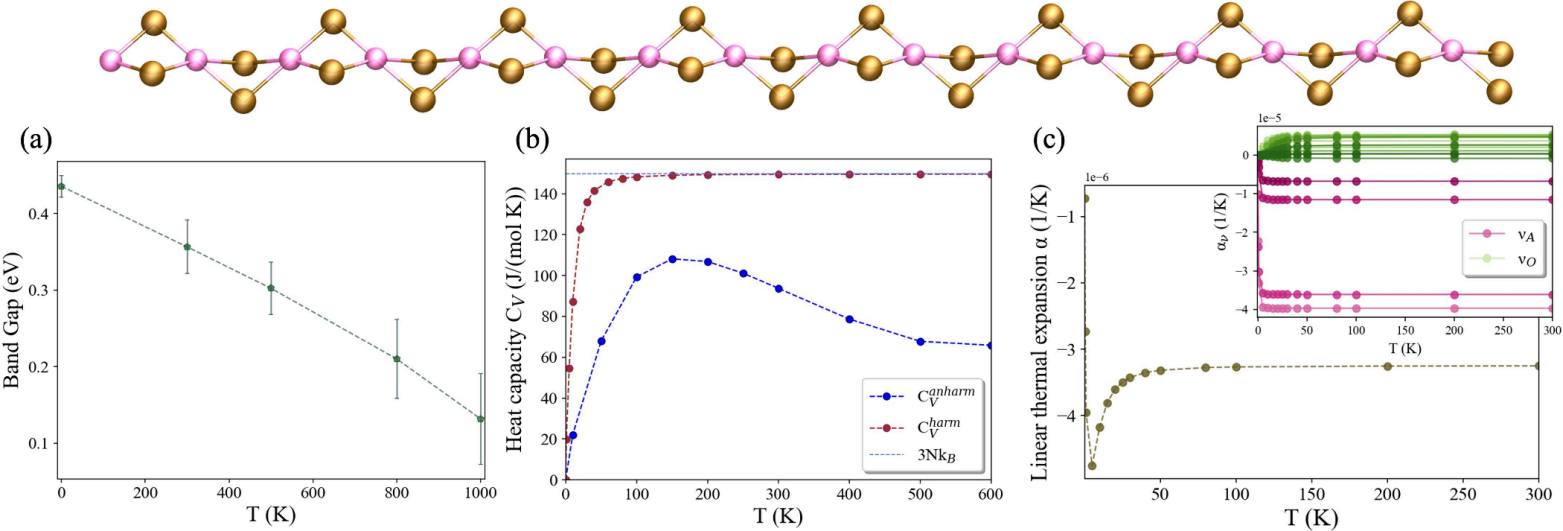
Au_2_Se_2_ (Au, pink; Se, brown). (a) Band-gap behavior with temperature in the stable insulating double-cell phase. (b) Anharmonic heat capacity calculated within the SSCHA with eq 2 (blue line) and harmonic heat capacity (brown line) using eq 3 with a temperature-independent harmonic DFPT ω. (c) Linear thermal expansion coefficient α­(*T*) using the QHA. In the inset, α­(*T*) per phonon branch. A and O are within the same group of modes, and lighter colors correspond to lower frequencies.

We report the anharmonic heat capacity of AuSe_2_ (Figure 3b) and its comparison with the 3*Nk*_B_ limit: same as the previous cases, AuSe_2_ reveals considerable anharmonicity, with a strong difference between *C*_*V*_^harm^ and *C*_*V*_^anharm^ already at small temperatures. The high-temperature limit of the heat capacity (see the SI) shows a similar decreasing trend to TaSe_3_ with a nonlinearity ω variation even stronger than CuC_2_, which makes the heat capacity diverge from the Dulong–Petit law.

The AuSe_2_ minimum linear thermal coefficient using the SSCHA is α = (−7.10 ± 0.25) × 10^–5^ K^–1^ (see Figure 4 and the SI). This value, as in CuC_2_, outlines a giant thermal contraction, superior to all other materials showing NTE. In Figure 3c, the QHA results for α are presented. The temperature dependence resembles that observed in other materials such as graphene and CNTs,^48,49^ but here we find a much sharper decrease, with the maximal contraction occurring at extremely low temperatures. As before, the QHA results give just a qualitative understanding of this trend. We recognize that the four acoustic phonons are responsible for the contraction (visible in the inset), while optical phonons instead present smaller and positive α. All acoustic eigenmodes ν_A_ correspond to Se oscillations along *x̂* and *ŷ*, causing bending or twisting of the wire, with minor movements of Au along the same transversal direction.

**Figure 4:**
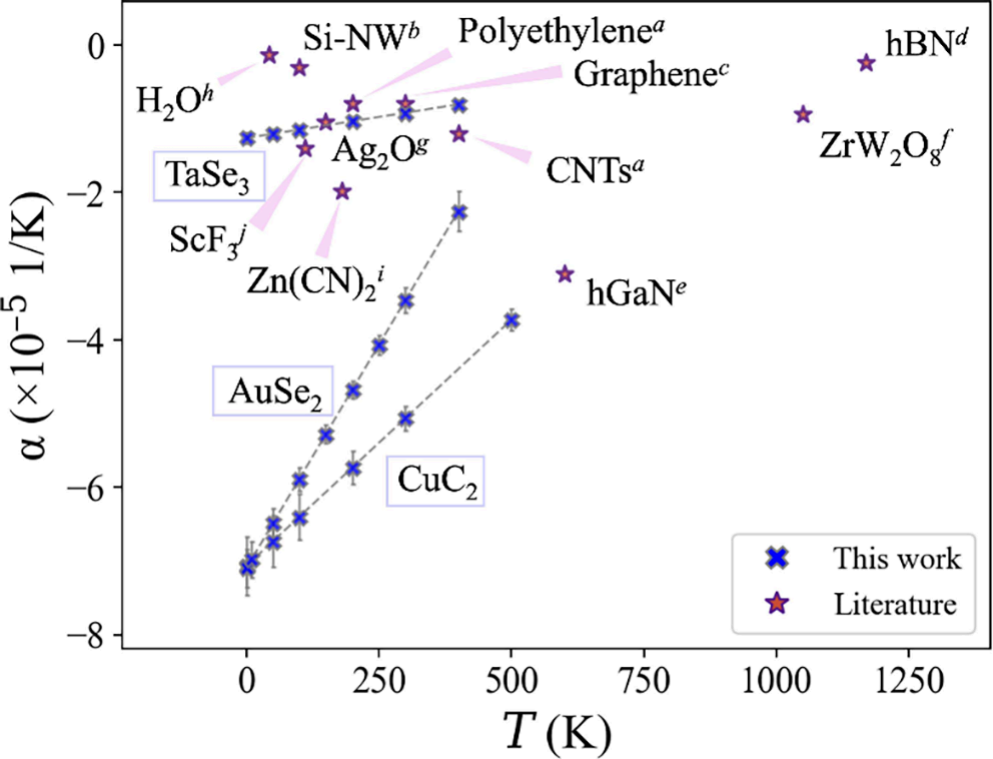
Comparison of the NTE coefficients for different materials: (a) ref 49; (b) ref 59; (c) refs^60^ and^66^; (d) ref 67; (e) ref 68; (f) refs^69^ and^70^; (g) ref 71; (h) refs 74 and 75; (i) ref 76; (j) ref 72. We plot the highest α value found for the material at the corresponding temperature. In the blue points, the temperature behavior of α for the three wires is studied in this work using the SSCHA and eq 1 (more details can be found in the SI).

In summary, we investigated the thermodynamic properties of 1D wires employing the SSCHA to analyze the thermal stability, heat capacity, and thermal expansion, fully accounting for quantum and anharmonic effects. Our results uncover thermodynamic behaviors that are common across all three materials studied, despite their diverse characteristics (a metal, a high-temperature unstable system, and an insulator with CDW), suggesting that our findings are general features of 1D materials, further supported by similar observations in CNTs and cumulene.

We observe extremely strong anharmonic behavior, underscoring the importance of anharmonicity in the thermodynamics of 1D systems.

All three wires exhibit extremely large NTE. Materials that contract with temperature are relatively rare but extremely useful in applications, for example, to compensate for the thermal expansion in composite materials. NTE has been observed in quasi-1D polymers, CNTs, or nanowires,^49,58,59,65^ in layered materials such as graphene and 2D nitrides,^48,60,66–68^ and in 3D crystals like cubic ZrW_2_O_8_ and related compounds, ZrV_2_O_7_ family, ScF_3_, and cuprite structures.^56,69–72^ We also mention that the 3D compound Ag_3_[Co­(CN)_6_][Bibr ref73] exhibits an anisotropic thermal expansion coefficient α_*c*_ of −12 × 10^–5^ *K* ^–1^ along the *c* axis and positive thermal expansion along the other two axes (in contrast to the isotropic contraction observed in previously mentioned NTE materials and resulting in an overall positive thermal expansion). A comparison of the α coefficients for different known NTE materials is presented in Figure 4. As is visible, CuC_2_ and AuSe_2_ stand out as the greatest contractions among all.

We noted that, in all three cases, the contraction is due to a softening of the vibrational modes (mostly acoustic) along the *x̂*–*ŷ* perpendicular directions of the wire, typically twisting and bending the wires. This behavior is observed in other low-dimensional systems^48,49^ and has been studied in various 2D materials.^46,66,67^ Modulation of the monolayer in the transversal directions gives rise to ripples^66^ predicted by Liftshitz in 1952 ^77^ and known as the *membrane effect*.^48^

In conclusion, 1D materials offer significant promise for technological applications, and the three materials analyzed here are notable examples. This study demonstrates that all of the wires analyzed show remarkable thermodynamic properties. A systematic exploration that rigorously accounts for anharmonicity could uncover additional outstanding candidates for future applications.

## Supplementary Material



## Data Availability

The implemented ASRs for 1D materials are made available on GitHub as part of the python-sscha distribution.^45^ The AiiDA interface used to handle the explicit DFT calculations is available through the AiiDAEnsemble class, from SSCHA version 1.4.0.^78^ The trained force fields for the three wires are freely available and can be found on the Materials Cloud archive,^79^ together with all of the relevant input and output files.^79^

## Notes

Detailed computational methods, with parameters employed in the calculations; details on the calculations of the linear thermal expansion coefficient, with components of the SSCHA stress tensor along the wire as a function of the temperature for the three materials, together with a discussion on error sources; high-temperature limit of the specific heat capacities for CuC_2_, TaSe_3_, and AuSe_2_ with a discussion; TaSe_3_ unstable structures following the unstable modes at high temperature; details on the training of the machine-learning potentials, with a discussion of ML errors, and a comparison of harmonic phonon dispersions between ML and explicit DFPT calculations (PDF)
