# Supplementary material for: Extreme Anharmonicity and Thermal Contraction of One-Dimensional Wires
Source: Nano Lett. 2025 Oct 27;25(44):15919–26. doi: 10.1021/acs.nanolett.5c04282 (PMC12593410; doi:10.1021/acs.nanolett.5c04282)
Supplement: Supplementary file 1 [file nl5c04282_si_001.pdf]

Supporting Information for: Extreme Anharmonicity and Thermal Contraction of One-Dimensional Wires

Chiara Cignarella,[*,†,‡] Lorenzo Bastonero,[‡] Lorenzo Monacelli,[¶] and Nicola Marzari[†,§,‡]

†*Theory and Simulation of Materials (THEOS), and National Centre for Computational Design and Discovery of Novel Materials (MARVEL), École Polytechnique Fédérale de Lausanne, 1015 Lausanne, Switzerland*

‡*U Bremen Excellence Chair, Bremen Center for Computational Materials Science, and MAPEX Center for Materials and Processes, University of Bremen, 28359 Bremen, Germany*

¶*Departement of Physics, Sapienza University of Rome, 00185 Rome, Italy*

§*PSI Center for Scientific Computing, Theory, and Data, Laboratory for Materials Simulations (LMS), Paul Scherrer Institut, 5232 Villigen, Switzerland*

E-mail: ccignare@uni-bremen.de

The supporting information contains:

- **Computational methods**: description of the methods used to produce the results of the paper, with details and parameters employed in the calculations.
- **Linear thermal expansion coefficient and stress tensors**: component of the SSCHA stress-tensor along the wire in function of temperature for the three materials,

together to a discussion on the calculations of the linear thermal coefficients and error sources.

- **High-temperature limit of the heat capacity**: the high-temperature limit of the specific heat capacity for CuC_2, $TaSe_3$, and $AuSe_2$ with discussion .
- **$TaSe_3$ unstable modes**: $TaSe_3$ unstable structures following the unstable modes at high temperature.
- **Neural network potentials**: details on the training of the machine learning potentials, with discussion of ML errors and comparison of harmonic phonon dispersions between ML and explicit DFPT calculations.

All the relevant input and output files, and the machine learning potentials used to produce the results of this publication are freely available on the Materials Cloud Archive.[1]

Computational methods

The stocastic self-consistent harmonic approximation is a stocastic method which determines the ionic quantum free energy $F = U - TS$, (where U is the internal energy, T the temperature ans S the entropy) through the variational principle, by optimizing a trial ionic density matrix $\tilde{\rho}(\mathbf{R})$ to minimize F. The approximation consists in constraining the trial density matrix $\tilde{\rho}(\mathbf{R})$ to a Gaussian distribution defined by two parameters: the centroids $\mathcal{R}$, representing the average position of atoms in the system, and the Gaussian covariance matrix Φ, representing the quantum and thermal fluctuations of ions around the centroids. The Gaussian constraint on $\tilde{\rho}(\mathbf{R})$ results in an analytical expression for the entropy S that allows for a direct calculation and minimization of the free energy. The internal energy U, average forces, and stresses are evaluated stochastically: an ensemble of n random atomic configurations (referred to as *population*) is generated based on the $\tilde{\rho}(\mathbf{R})_{trial}$ probability in a chosen supercell, and energies, forces and stress tensors for each configuration are computed

by using *ab initio* calculations (e.g., density-functional theory). For more details on the method, we refer the reader to Ref. 2. To assess structural stability and the temperature-dependent anharmonic phonon spectrum, we define a positional free energy function $F(\mathcal{R})$, which depends on the ionic coordinates $\boldsymbol{R}$. This free energy is obtained by performing the SSCHA minimization while constraining the average ionic positions (centroids) to remain fixed at $\mathcal{R}$, optimizing only the force constant matrix Φ. This construction matches the free energy landscape introduced in Landau theory of phase transitions. The Hessian of $F(\mathcal{R})$ evaluated around the high-symmetry configuration determines both the structural stability and the temperature-dependent anharmonic phonons.[3,4]

In 1D materials, difficulties arise from the intrinsically low-frequency nature of their phonon dispersions, which hampers the conditioning of the free energy minimization as phonon frequencies approach zero or become very small. This effect is particularly significant in the calculation of the anharmonic phonon dispersion (the Hessian of the free energy), which diverges as $1/\omega^3$ for $\omega \to 0$, leading to highly noisy results.[5] We address the additional zero-frequency mode at Γ, which originates from the fourth acoustic rotational mode, by implementing the 1D acoustic sum rule within the SSCHA framework. Nevertheless, the slow convergence demands thousands of configurations per population, resulting in a prohibitive number of *ab initio* calculations. To overcome this, we train three machine-learning force fields, one for each wire.

To train these models, we use total energies, forces, and stresses from DFT electronic ground-state calculations performed on a number of frames extracted within SSCHA. The DFT explicit calculations are run with the open-source Quantum ESPRESSO distribution.[6] We employ the PBE functional[7] for the exchange-correlation term and pseudopotentials from the SSSP v1.1 PBE efficiency[8]. The sampling of the Brillouin zone is set to 1x1x60 for CuC_2 and 0.1 Ry of *cold* smearing, 1x1x20 for $TaSe_3$ with a smearing of 0.2 Ry, and 1x1x20 for $AuSe_2$ in the single unit cell and smearing of 0.2 Ry. The systems are inserted in ~ 25 Å of vacuum in the two perpendicular directions to the wire axis, in order to remove inter-

actions with the periodic images.[9] The calculations are handled by the automated workflow manager AiiDA[10,11] using the `aiida-quantumespresso` plugin interfaced for this work with the SSCHA code. The models are trained using the open-source NequIP code.[12] Details on the generation of the training dataset and the performance of the resulting machine-learning potentials are discussed in the Section *Neural network potentials.*

Initial guesses for the SSCHA minimization are provided by harmonic phonons computed using DFPT.[13] SSCHA calculations are performed on supercell sizes of 1x1x6 for CuC_2 and $TaSe_3$, and 1x1x10 for the stable phase of $AuSe_2$. The fourth-root representation has been employed to ensure the dynamical matrix is always positive during the minimization.[5] Finally, we apply the interpolation scheme and acoustic sum rule as implemented in Ref. 14 on top of the final minimized Hessian.

For the calculation of the linear thermal expansion coefficients, we use eq. (1) of the main text with the Young's modulus Y for assessing β_T, taken at zero temperature from Ref. 15. The constant-volume derivative of the pressure is evaluated from finite differences of the anharmonic stress tensor along the axial direction computed through the SSCHA.[5] From these data, we evaluate the α behavior with temperature presented in Fig. 4 of the main text, assuming a weak temperature-dependence of the Young modulus for low-temperatures. A more comprehensive description of these calculations are given in the next Section *Linear thermal expansion coefficient and stress tensors*, together to a discussion on error sources.

The α coefficients within the QHA are calculated in terms of the Grüneisen γ parameter:

$$\alpha = \frac{1}{\frac{\partial^2 E}{\partial s^2}} \sum_{\mathbf{q},\mu} c_v(\mathbf{q},\mu)\gamma(\mathbf{q},\mu), \tag{1}$$

where E is the electronic energy at different strains, $c_v(\mathbf{q},\mu)$ is the harmonic heat capacity of individual phonon bands defined by eq. (3) of the main text, and $\gamma(\mathbf{q},\mu)$ is defined for

harmonic phonon bands of energy $\omega_{\mathbf{q},\mu}$ as:

$$\gamma(\mathbf{q},\mu) = -\frac{1}{2\omega^0_{\mathbf{q},\mu}}\frac{\partial \omega_{\mathbf{q},\mu}}{\partial s}, \tag{2}$$

where $s = (a - a_0)/a_0$ is the tensile strain.

To obtain the $AuSe_2$ energy gap at different temperatures, we compute E_g as the energy difference between LUMO and HOMO for 6 random configurations extracted from the minimized SSCHA ensemble, for every temperature considered. The resulting band gap at each temperature is taken as the average of these 6 values. The associated error is computed as the standard deviation of this average. Systematic sources of errors are expected to be the same across all temperatures and therefore should not affect the trend, as discussed in next section *Linear thermal expansion coefficient and stress tensors*

Linear thermal expansion coefficient and stress tensors

We report the stress tensor component along the wire axis $\hat{z}$ for the three materials under study, calculated using constant-volume SSCHA calculations at various temperatures. These values are normalized by the actual volume of each wires. In fact, simulations of one-dimensional materials in periodic boundary conditions require a vacuum space to remove spurious interactions between adjacent replicas, and therefore the wire volume differs from the cell volume used in calculations. For the former, we adopt the definition of the quantum volume,[16] calculated for the three systems in Ref. 15. In Fig. 1 the results are shown, along with a quadratic interpolation of $P_{zz}(T)$, which we found to fit well our data.

The linear thermal expansion coefficient at T = 0 K is obtained from its slope, divided by the Young's modulus computed at 0 K in Ref. 15. The primary source of error in this calculation is the stochastic error from the fit, which represents the 5.5 %, 5.9 % and 3.6 % respectively for CuC_2, $TaSe_3$ and $AuSe_2$. Other sources of error are represented by the SCHA approximation, the machine learning force fields and the PBE functional used to

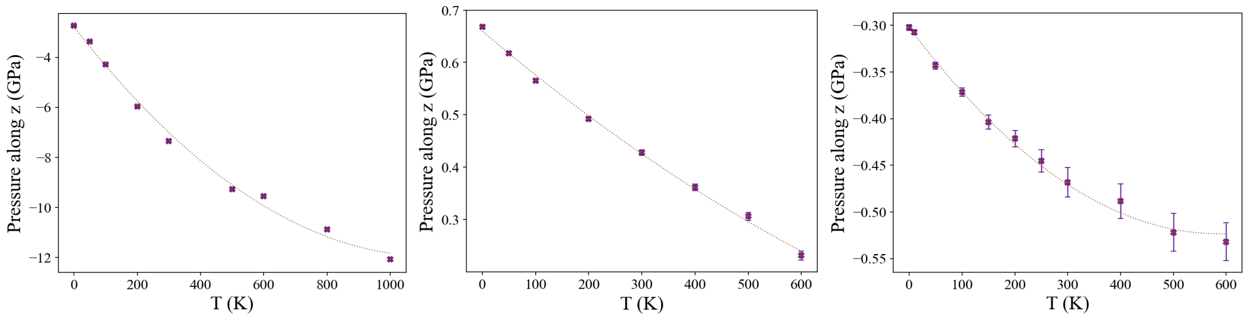

(a) (b) (c)

Figure 1: Stress tensor component along the wire axis, from left to right, for (a) CuC_2, (b) $TaSe_3$, and (c) $AuSe_2$. In dashed line the quadratic interpolation.

train the potentials. We expect these systematic errors to be relatively small and similar for all temperatures; therefore, their impact on the temperature-dependent trends and the derivative quantities is likely to be minor.

The temperature-dependent α in Fig. 4 of the main text are obtained from the derivative of the quadratic fit and using the Young's modulus at 0 K, with the assumption of weak Y temperature-dependence. We estimate the variation of the Young's modulus with temperature using QHA, resulting less than ~ 0.5 %, 1.5 %, and 4 % for CuC_2, $TaSe_3$, and $AuSe_2$ respectively, at T $= 300$ K, which indicates that the error committed with this assumption is small, and does not change both the qualitative and quantitative results. For CuC_2 and $TaSe_3$ it remains negligible (~ 0.6 % and 2 %) up to T $= 500$ K, while 5.4 % at 400 K for $AuSe_2$.

From these calculations, we observe only a (linear) rise of $\alpha(T)$, but no decrease, as in CNTs or graphene. This can be understood looking at the QHA results in the main paper, Figures 1 (c), 2 (c), 3 (c) , where the minimal contraction followed by the upturn of α occurs at extremely low temperature (even below 1 K for $TaSe_3$). Such steep variation, in the range of T $\approx$ 0 - 5 K, is not sampled by our SSCHA calculations, as they are performed with a larger temperature step. Therefore, the trend shown in Fig. 4 of the main text reflects only the rising range, which lies in the relevant temperature range for electronic applications.

High-temperature limit of the heat capacity

At high-temperature, when T is greater than the Debye temperature T_D, the constant-volume heat capacity C_V of classical harmonic crystals obeys the Dulong-Petit law: $\lim_{T\to\infty} C_v^{harm} = 3Nk_B$. Anharmonic effects are responsible for deviations of the specific heat from this constant value at high temperatures.[17–19] Eq. (2) in the main text describes the fully-anharmonic heat capacity, where, in this limit, the harmonic part c_v^{harm} reduces to the Dulong-Petit constant value, and the heat capacity can be written as:

$$C_V(T) \overset{\mathrm{T} \gg \mathrm{T_D}}{=} 3Nk_B + \sum_{\mathbf{q},\mu} \frac{k_B T}{\omega_{\mathbf{q},\mu}} \left(\frac{\partial \omega_{\mathbf{q},\mu}}{\partial T} \right)_V . \tag{3}$$

The last term highlights the deviation from the classical harmonic behavior due to anharmonic effects.

In Fig. 2, we present the heat capacity in this limit, computed using eq. (3) for the three wires, which quantifies the degree of anharmonicity in one-dimensional wires.

In all cases, the last term in eq. (3) results in net negative contribution, and its large variation underlines again a strong anharmonicity. For instance, the decrease in C_V with temperature surpasses that observed in $CsSnI_3$ in Ref. 20, which is considered a highly anharmonic 3D system.

The trend observe for CuC_2 (Fig. 2(a)) originates from the competition between the softening of high-frequency optical modes and the hardening of low-frequency phonon modes upon heating (also visible from the phonon dispersion in the main text, Fig. 1(a)). On the other hand, for $TaSe_3$, all the frequencies decrease as the temperature rises, leading to an overall decreasing of the anharmonic C_V term with T, in Fig. 2(b). Finally, we notice that anharmonicity strongly affects the total heat capacity in $AuSe_2$ (Fig. 2(c)), which varies the most among the three wires, and due to a strong non-linear dependence of the phonon frequencies on temperature.

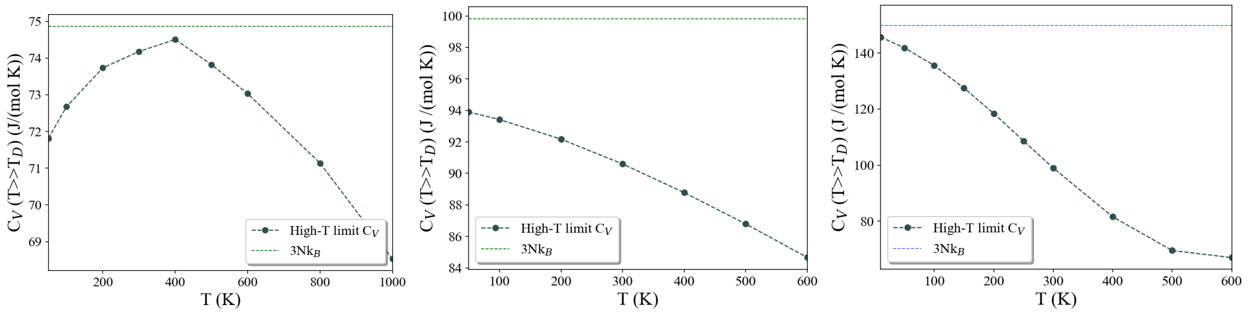

(a) (b) (c)

Figure 2: High-temperature limit of the heat capacity, as given by eq. (3), and in green, the Dulong-Petit constant value of classical harmonic crystals. From left to right: (a) CuC_2, (b) $TaSe_3$, and (c) $AuSe_2$

.

$TaSe_3$ unstable modes

We report in Figure 3 the distorted structural configurations of $TaSe_3$ wire following the two unstable acoustic modes at T=1000 K, visible in the phonon dispersion and indicated by the blue arrow in Fig. 2(a) of the main text. The wire shows the tendency to distort along the perpendicular direction, with Se atoms moving asymmetrically and inducing a bending of the structure.

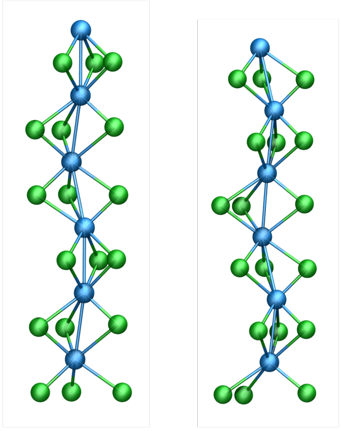

Figure 3: $TaSe_3$ unstable structures at high temperature following the first (left) and second (right) unstable acoustic mode (ν_{A_1} and ν_{A_2}).

Neural network potentials

We use the equivariant neural network (NN) interatomic potential implemented in NequIP.[12] For models training, explicit density-functional theory calculations are performed on various random configurations of the system in a supercell, as generated by SSCHA. The models are trained using energies, forces and stresses of ensembles at different temperatures ranging from 0 K to 1600 K. The datasets include 1588 configurations for CuC_2, 1800 for $TaSe_3$ and a total of 4620 for $AuSe_2$. (The dataset for $AuSe_2$ is subdivided into 3270 of configurations in its initial phase and 1350 frames in the stable double-cell phase). About 10 % of configurations are picked from the CuC_2 and $TaSe_3$ datasets to be used as *testing set*, and $\sim$ 15 % from $AuSe_2$. The remaining configurations are shuffled and randomly subdivided into 80 % for *training set* and 20 % for *validation* during the training process. The graph neural network architecture is composed of 4 interaction blocks, and the multiplicity of the feature vectors is set to 32. The number of basis functions for the radial part is 8, with a cut-off radius r_c of 5 Å. The radial functions are learned by a multi-layer perceptron network with 2 layers. The maximum degree for the spherical harmonics is $l = 3$. We use a learning rate is 0.005 and a batch size of 4.

The final quality of the models is evaluated with root mean squared error (RMSE) and mean absolute error (MAE) summarised in Table 1.

Table 1: Test errors of the Nequip models for the system under analysis. We report the errors on each of the models on training, validation and test sets.

	F (meV/Å)						E (meV)[1]					
	MAE			RMSE			MAE			RMSE		
	train	valid.	test	train	valid.	test	train	valid.	test	train	valid.	test
CuC_2	32	33	39	79	93	122	10	12	23	16	45	109
$TaSe_3$	13	13	17	17	18	29	4	7	5	5	9	14
$AuSe_2$	7	9	9	12	19	14	40	36	16	61	48	17

To further assess the performance of our models, we show in Fig. 4 the comparison between DFPT phonon dispersions and phonon dispersions computed by finite differences

using the models trained for the three materials under study. We show $AuSe_2$ both in the initial and final stable configuration. Both DFPT and Nequip results are computed with the same **q**-grid, successively applying the acoustic sum rules from Ref. 14. The little wing around Γ in $TaSe_3$ is an interpolation artefact and disappears when more **q**-points are used (or, equivalently, a bigger supercell).

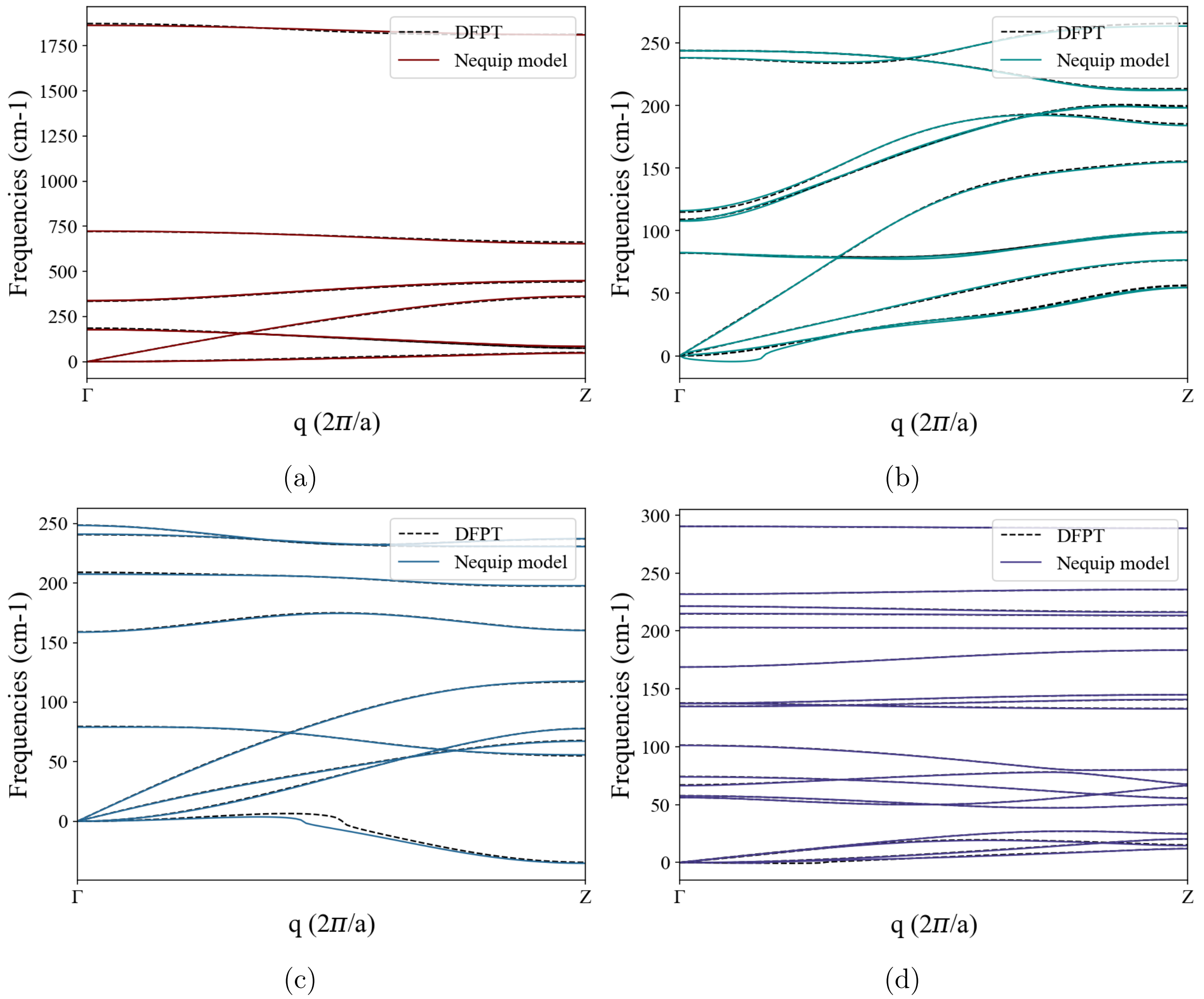

Figure 4: Comparison between DFPT and NequIP phonon dispersions for (a) CuC_2, (b) $TaSe_3$, and $AuSe_2$ in (c) initial, and (d) final configuration.

From both Table 1 and Figure 4 we observe that the models perform extremely well for all the materials considered, with maximum errors on forces of tens of meV/Å and exceptional agreement between DFPT and NequIP.

References

(1) Cignarella, C.; Bastonero, L.; Monacelli, L.; Marzari, N. Extreme anharmonicity and thermal contraction of 1D wires. *Materials Cloud Archive* **2025**, 2025.149, DOI: 10.24435/materialscloud:fj-20.

(2) Monacelli, L.; Bianco, R.; Cherubini, M.; Calandra, M.; Errea, I.; Mauri, F. The stochastic self-consistent harmonic approximation: calculating vibrational properties of materials with full quantum and anharmonic effects. *Journal of Physics: Condensed Matter* **2021**, *33*, 363001.

(3) Bianco, R.; Errea, I.; Paulatto, L.; Calandra, M.; Mauri, F. Second-order structural phase transitions, free energy curvature, and temperature-dependent anharmonic phonons in the self-consistent harmonic approximation: Theory and stochastic implementation. *Phys. Rev. B* **2017**, *96*, 014111.

(4) Monacelli, L. Simulating anharmonic crystals: Lights and shadows of first-principles approaches. *arXiv preprint arXiv:2407.03090* **2024**,

(5) Monacelli, L.; Errea, I.; Calandra, M.; Mauri, F. Pressure and stress tensor of complex anharmonic crystals within the stochastic self-consistent harmonic approximation. *Physical Review B* **2018**, *98*, 024106.

(6) Giannozzi, P.; Baroni, S.; Bonini, N.; Calandra, M.; Car, R.; Cavazzoni, C.; Ceresoli, D.; Chiarotti, G. L.; Cococcioni, M.; Dabo, I.; others QUANTUM ESPRESSO: a modular and open-source software project for quantum simulations of materials. *Journal of Physics: Condensed Matter* **2009**, *21*, 395502.

(7) Perdew, J. P.; Burke, K.; Ernzerhof, M. Generalized Gradient Approximation Made Simple. *Physical Review Letters* **1996**, *77*, 3865–3868.

(8) Prandini, G.; Marrazzo, A.; Castelli, I. E.; Mounet, N.; Marzari, N. Precision and efficiency in solid-state pseudopotential calculations. *npj Computational Materials* **2018**, *4*, 1–13.

(9) Kozinsky, B.; Marzari, N. Static Dielectric Properties of Carbon Nanotubes from First Principles. *Phys. Rev. Lett.* **2006**, *96*, 166801.

(10) Huber, S. P.; Zoupanos, S.; Uhrin, M.; Talirz, L.; Kahle, L.; Häuselmann, R.; Gresch, D.; Müller, T.; Yakutovich, A. V.; Andersen, C. W.; others AiiDA 1.0, a scalable computational infrastructure for automated reproducible workflows and data provenance. *Scientific data* **2020**, *7*, 300.

(11) Uhrin, M.; Huber, S. P.; Yu, J.; Marzari, N.; Pizzi, G. Workflows in AiiDA: Engineering a high-throughput, event-based engine for robust and modular computational workflows. *Computational Materials Science* **2021**, *187*, 110086.

(12) Batzner, S.; Musaelian, A.; Sun, L.; Geiger, M.; Mailoa, J. P.; Kornbluth, M.; Molinari, N.; Smidt, T. E.; Kozinsky, B. E(3)-equivariant graph neural networks for data-efficient and accurate interatomic potentials. *Nature Communications* **2022**, *13*, 2453.

(13) Baroni, S.; De Gironcoli, S.; Dal Corso, A.; Giannozzi, P. Phonons and related crystal properties from density-functional perturbation theory. *Reviews of Modern Physics* **2001**, *73*, 515, Publisher: APS.

(14) Lin, C.; Poncé, S.; Marzari, N. General invariance and equilibrium conditions for lattice dynamics in 1D, 2D, and 3D materials. *npj Computational Materials* **2022**, *8*, 236.

(15) Cignarella, C.; Campi, D.; Marzari, N. Searching for the thinnest metallic wire. *ACS nano* **2024**, *18*, 16101–16112.

(16) Cococcioni, M.; Mauri, F.; Ceder, G.; Marzari, N. Electronic-enthalpy functional for finite systems under pressure. *Physical Review Letters* **2005**, *94*, 145501.

(17) Peierls, R.; Peierls, R. E. *Quantum theory of solids*; Oxford University Press, 1955; Chapter II.

(18) Brüesch, P. *Phonons: Theory and experiments I: Lattice dynamics and Models of interatomic forces*; Springer Science & Business Media, 2012; Vol. 34; Chapter 5.

(19) Ashcroft, N. W.; Mermin, N. *Solid state*; 1976; Chapter 25.

(20) Monacelli, L.; Marzari, N. First-principles thermodynamics of CsSnI3. *Chemistry of Materials* **2023**, *35*, 1702–1709.
